# Comparison between Bioactive Sol-Gel and Melt-Derived Glasses/Glass-Ceramics Based on the Multicomponent SiO_2_–P_2_O_5_–CaO–MgO–Na_2_O–K_2_O System

**DOI:** 10.3390/ma13030540

**Published:** 2020-01-23

**Authors:** Elisa Fiume, Carla Migneco, Enrica Verné, Francesco Baino

**Affiliations:** 1 Institute of Materials Physics and Engineering, Department of Applied Science and Technology, Politecnico di Torino, 10129 Turin, Italy; s253000@studenti.polito.it; 2 Department of Mechanical and Aerospace Engineering, Politecnico di Torino, 10129 Turin, Italy; 3 Interdepartmental Centre PoliTo BIOMedLab, Politecnico di Torino, 10129 Turin, Italy; 4 Interdepartmental Centre J-Tech@PoliTO, Politecnico di Torino, 10129 Turin, Italy

**Keywords:** bioactive glass, glass-ceramic, biomaterials, bioceramics, sol-gel, bioactivity, porosity, bone tissue engineering, textural properties, nanomaterials

## Abstract

Bioactive sol-gel glasses are attractive biomaterials from both technological and functional viewpoints as they require lower processing temperatures compared to their melt-derived counterparts and exhibit a high specific surface area due to inherent nanoporosity. However, most of these materials are based on relatively simple binary or ternary oxide systems since the synthesis of multicomponent glasses via sol-gel still is a challenge. This work reports for the first time the production and characterization of sol-gel materials based on a six-oxide basic system (SiO_2_–P_2_O_5_–CaO–MgO–Na_2_O–K_2_O). It was shown that calcination played a role in inducing the formation of crystalline phases, thus generating glass-ceramic materials. The thermal, microstructural and textural properties, as well as the in vitro bioactivity, of these sol-gel materials were assessed and compared to those of the melt-derived counterpart glass with the same nominal composition. In spite of their glass-ceramic nature, these materials retained an excellent apatite-forming ability, which is key in bone repair applications.

## 1. Introduction

Bioactive glasses are commonly considered eligible materials for bone tissue engineering applications as they are able to promote bone tissue healing [1,2,3] due to a progressive dissolution process, which releases into the physiological environment ionic products able to stimulate osteoblastic activity and, thus, the growth of new tissue [4,5].

As a result, bioactive glasses have been extensively investigated for the treatment of medium-to-critical sized bone defects deriving from chronical diseases and traumatic events [6,7,8]. According to the final clinical application, bioactive glasses can be processed in the form of monoliths, particles and porous three-dimensional (3D) scaffolds mimicking the trabecular bone architecture [9]. However, current clinical approaches for the treatment of small defects in non-bearing anatomical sites mainly rely on the usage of granules and particles due to a paucity of international rules concerning the manufacturing and validation processes of bioactive glass-based porous 3D scaffolds [10,11].

The first use of bioactive glass particles in the clinical practice dates back to 1993, when Hench’s 45S5 Bioglass was marketed under the tradename of PerioGlas for the treatment of periodontal diseases, with particles diameter in the range of 90–710 μm [9]. Compared to large-size cast products and monoliths or porous scaffolds obtained from thermal consolidation of glass powder (sintering), bioactive glass particles generally expose a much higher specific surface area (SSA), which leads to higher conversion rate into hydroxyapatite: this is key in all those applications where a fast new bone deposition is required. The strategies commonly adopted to further improve the reaction kinetics of glasses in the physiological environment rely on the appropriate compositional design, method of production and additional treatments (e.g., calcination conditions). It is known, for example, that bioactive glass powders can be produced by both a traditional melt-quenching route and the sol-gel process [12,13]. Most melt-derived bioactive glasses, however, are affected by a series of limitations. Melt-quenching requires high temperatures (typically around 1500 °C) to allow the melting of the oxide precursors. Moreover, bioactivity in melt-derived silicate glasses is possible only in a limited compositional range, as SiO_2_ amounts higher than 60 mol % make the material almost chemically inert in contact with body fluids [3].

Compared to the traditional melt-quenching route, the sol-gel process offers the possibility to obtain more reactive materials in a wider compositional range (up to 90% of SiO_2_) due to the unique textural properties (inherent nanoporosity) that directly derive from the synthesis process [14,15,16,17,18]. The 45S5 Bioglass was produced via both melt-quenching [3] and sol-gel routes [19,20,21] but, to date, the latter strategy has been seldom applied. In this regard, the first attempt was reported by Chen and Thouas [19] who first succeeded in introducing Na_2_O in the sol-gel synthesis of 45S5 bioactive glass. Later, Cacciotti and coworkers [20] demonstrated that the crystallinity, bioactive mechanism and reaction kinetics in vitro of sol-gel 45S5 glass can be modulated by appropriate post-synthesis thermal treatments. In another study, Faure et al. [21] reported the sol-gel synthesis of 45S5 bioactive glass by using an organic acid as a catalyst. A slight increase of the SSA was observed in the sol-gel material with respect to the melt-derived counterpart (from 0.4 m^2^/g to 0.9 m^2^/g), while the apatite-forming ability was found to be comparable to that of commercial cast 45S5 Bioglass.

A review of the literature shows that most of the sol-gel glasses belong to binary (SiO_2_–CaO) or ternary systems (SiO_2_–CaO–P_2_O_5_) [17,22,23] with high amounts of silica; as discussed above, the 45S5 composition is one of the very few “quaternary” exceptions [19,20,21]. Unlike melt-derived glasses, it is not strictly necessary to include too high of an amount of modifier oxides to lower the processing temperature of gel-derived glasses since the formation of the glass network occurs at room temperature [9]. However, some additional elements are highly beneficial for tailoring the bioactive and even biological response of the material (therapeutic effect). For example, CaO and MgO were reported to play an important role in surface reaction kinetics, new bone formation and bone cell adhesion and stability [24,25,26]. Silver was also incorporated in sol-gel SiO_2_–CaO glasses to obtain foam-like scaffolds with antibacterial properties [27].

In this work, we applied, for the first time, the sol-gel process to synthesize bioactive materials based on a complex six-oxide system. The parent SiO_2_–P_2_O_5_–CaO–MgO–Na_2_O–K_2_O composition was previously designed and investigated by our research group to produce a melt-derived glass (47.5B) [28,29], which was particularly appreciated because of its wide workability window and bioactive properties. Apart from innovatively describing the synthesis of this multicomponent glass by the sol-gel method, the present work also is one of the few available studies reporting a direct comparison between melt-derived and sol-gel materials with the same nominal composition.

## 2. Materials and Methods

### 2.1. Production of Glass and Glass-Ceramic Materials

#### 2.1.1. Melt-Quenching Route

A 47.5B bioactive silicate glass with composition 47.5SiO_2_–20CaO–10MgO–2.5P_2_O_5_–10K_2_O–10Na_2_O (mol %) was produced by the traditional melt-quenching route as previously described by Fiume et al. [30]. Briefly, a blend of oxides and carbonates was heated in a capped platinum crucible up to 1000 °C (heating rate 12 °C/min) to allow the thermal decomposition of glass precursors. After that, the crucible cap was removed and the temperature inside the furnace was increased to 1500 °C (heating rate 15 °C/min). After 30 min, the melt was poured into distilled water to obtain a glass frit which was then left to dry at room temperature for 24 h. Glass powders were obtained by ball milling (Pulverisette 0, Fritsch, Idar-Oberstein, Germany) and sifted (stainless steel sieve, Giuliani Technology Srl, Turin, Italy) in order to get a final particle size below 32 µm. Melt-derived 47.5B bioactive glass, hereafter named MD-47.5B, was used as a control in the present study.

#### 2.1.2. Sol-Gel Synthesis

Multicomponent materials based on the six-oxide system 47.5SiO_2_–20CaO–10MgO–2.5P_2_O_5_–10K_2_O–10Na_2_O (mol %) were produced by the sol-gel process for the first time. Tetraethyl orthosilicate (TEOS), tri-ethyl phosphate (TEP), calcium nitrate tetrahydrate (Ca(NO_3_)_2_·4H_2_O), sodium nitrate (NaNO_3_), magnesium nitrate hexahydrate ((MgNO_3_)_2_·6H_2_O) and potassium nitrate (KNO_3_) were used as SiO_2_, P_2_O_5_, CaO, Na_2_O, MgO and K_2_O sources, respectively. A solution comprising 10 mL HNO_3_ (2N) and 60 mL distilled water was mixed in sealed flasks for 5 min at room temperature; the acid served as a catalyst for the subsequent hydrolysis of TEOS. Afterward, TEOS was added to the solution that was mixed under continuous magnetic stirring (200 rpm) for 15 min. An H_2_O:TEOS molar ratio of 20 was used in good accordance with the study reported by Bahniuk et al. [31]. All the other reagents were then sequentially added to the batch, which was mixed for 45 min until a clear and homogeneous sol was obtained.

Gelation was carried out at room temperature for 72 h. After that, samples were aged for 72 h in an oven at 60 °C. The treatment was performed by maintaining the flasks sealed in order to prevent the dispersion of volatile components. For the drying treatment, the flasks were slightly opened to allow the slow evaporation of the alcoholic liquid phase while the temperature was increased up to 120 °C (48 h).

**Figure 1:**
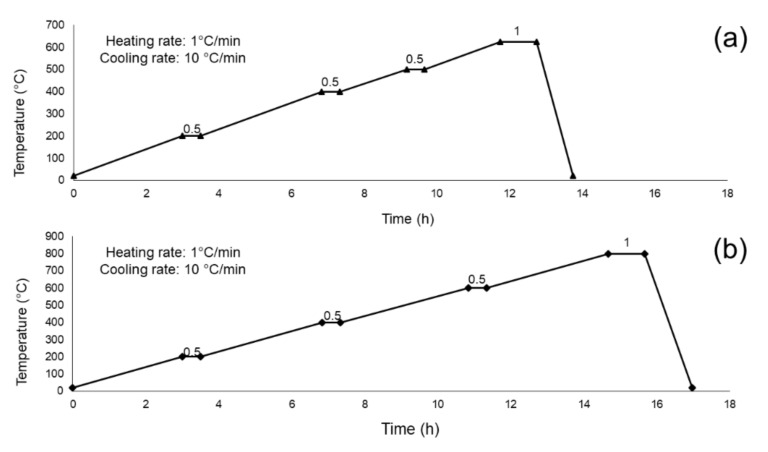
Heating programs used for the calcination of DG-120 at T_s1_ = 625 °C (**a**) and T_s2_ = 800 °C (**b**). Intermediate dwelling times and final calcination temperatures were identified on the basis of the differential thermal analysis results.

In order to follow the evolution of the material, part of the dried gel was milled and stored in a drier closet to avoid moisture absorption (sample DG-120), while the remaining one was calcined up to 625 °C (T_s1_) (sample SG-625) or 800 °C (T_s2_) (sample SG-800), following the two heating programs displayed in Figure 1. The calcined materials (SG-625 and SG-800) were then ball milled and sieved (mesh 32 μm), as previously described for the MD-47.B system.

### 2.2. Materials Characterizations

#### 2.2.1. Thermal Analyses

Differential thermal analysis (DTA; DTA404PC, Netzsch, Selb, Selb, Germany) was performed on MD-47.5B and DG-120 samples to investigate and compare the thermal behaviors of both materials, which had the same nominal oxide composition but were produced by melting or the sol-gel process; specifically, glass transition temperature (T_g_), crystallization onset temperature (T_x_) and maximum rate of crystallization temperature (T_c_). Furthermore, this analysis was useful to select the calcination temperatures of sol-gel DG-120. For the analysis, MD-47.5B and DG-120 powders (50 mg) were heated up to 1200 °C (heating rate of 10 °C/min) in platinum crucibles, using high-purity Al_2_O_3_ alumina powder as reference material. Under the same conditions and using the same equipment, thermogravimetric analysis (TGA) was concurrently performed on DG-120 to quantify the mass loss of the material upon heating.

The analysis of the DTA plots was carried out according to the following criteria:

- T_g_ was identified at the inflection point, as obtained from the first derivative of the plot;
- Peaks in the positive verse of the y-axis (maxima) were associated to exothermal reactions, while peaks in the negative verse of the y-axis (minima) were attributed to endothermal reactions.

#### 2.2.2. X-Ray Diffraction (XRD)

X-ray diffraction analysis (XRD; X’Pert Pro PW3040/60 diffractometer, PANalytical, Eindhoven, Netherlands) was performed on MD-47.5B, DG-120, SG-625 and SG-800 powders to assess the microstructural features of the various materials and identify the presence of crystalline phases deriving from the thermal treatment. The analysis was performed using a Bragg–Brentano camera geometry with a Cu Kα incident radiation (wavelength λ = 0.15405 nm). The 2θ angle was varied in a range of 10°–70°; voltage and current were fixed at 40 kV and 30 mA, respectively. Step counting time for data acquisition was set at 1 s with a step size of 0.02°. Powder size for SG-625 and SG-800 was below 32 µm. Crystalline phases were identified by using X’Pert HighScore software 2.2b (PANalytical, Eindhoven, The Netherlands) equipped with the PCPDFWIN database.

#### 2.2.3. Pore Analysis

Nitrogen (N_2_) adsorption–desorption porosimetry (ASAP2020 Micromeritics, Norcross, GA, USA) was used to evaluate and compare the textural properties of the materials and to identify possible effects of the synthesis method. The SSA was assessed by applying the Brunauer–Emmett–Teller (BET) theory [32].

#### 2.2.4. In Vitro Bioactivity Tests

The apatite-forming ability of MD-47.5B, SG-625 and SG-800 powders was investigated by soaking the materials powders in a simulated body fluid (SBF, pH = 7.40 at body temperature), which was prepared following the protocol proposed by Kokubo and Takadama in 2006 [33].

A mass-to-volume (powder/SBF) ratio of 1.5 mg/mL was used, according to a previous study reported by the Technical Committee 4 (TC04) of the International Commission on Glass (ICG) [34]. In vitro bioactivity tests were performed at 37 °C in an orbital shaker incubator (IKA 3510001 KS 4000 I Control Incubator Shaker, IKA-Werke GmbH & Co. KG, Staufen, Germany), keeping constant the shaking speed at 100 rpm.

The pH was monitored at specific time points (6 h, 24 h, 48 h, 72 h, 168 h and 336 h) at 37.0 ± 0.01 °C in order to qualitatively evaluate the ion exchange between the material surface and the solution on the basis of the pH variations observed.

At the end of the experiment, the test tubes containing powders and SBF were placed into a centrifuge (Hermle Z306 Universal Certrifuge, Benchmark Scientific Inc., Edison, NJ, USA) to allow powders to decant at the bottom of the tube. Afterward, the SBF was completely removed by a syringe and the powders were rinsed with bi-distilled water. After water removal, the powders were left to dry at 37 °C in static conditions for 48 h. Once dried, the powders were stored into sealed plastic tubes (Eppendorf, Hamburg, Germany) before undergoing morphological and compositional evaluation.

#### 2.2.5. Morphological and Compositional Investigations

The morphology and composition of samples before (DG-120, SG-625 and SG-800) and after in vitro bioactivity tests (MD-47.5B, SG-625 and SG-800) were investigated by scanning electron microscopy (SEM) and energy-dispersive X-ray spectroscopy (EDS) (field-emission SEM equipped with EDS; Supra^TM^ 40, Zeiss, Oberkochen, Germany) in order to evaluate the surface evolution occurring as a result of the reaction process between the material and the solution upon soaking. For the analysis, powders were fixed onto a carbon adhesive tape and sputter-coated with a thin layer of chromium (7 nm). The inspection voltage was set at 15 kV.

## 3. Results and Discussion

MD-47.5B is a silica-based bioactive glass previously designed and characterized by our research group. The high amount of modifier oxides, along with the high Ca/P ratio, make this glass very reactive in the physiological environment and confer to the system an exceptional apatite-forming ability, which was demonstrated for both powders and 3D porous scaffolds [28,30,35,36]. Moreover, this melt-derived glass exhibits a wide workability window, which makes it an optimal candidate for scaffold manufacturing: in fact, it is possible to produce highly densified macroporous structures upon sintering at a wide temperature range without affecting the reactivity of the material [29].

The present study compares the MD-47.5B system with two properly designed sol-gel materials having the same nominal composition in order to evaluate the effect of the synthesis route on the bioactivity and the textural properties of the material.

**Figure 2:**
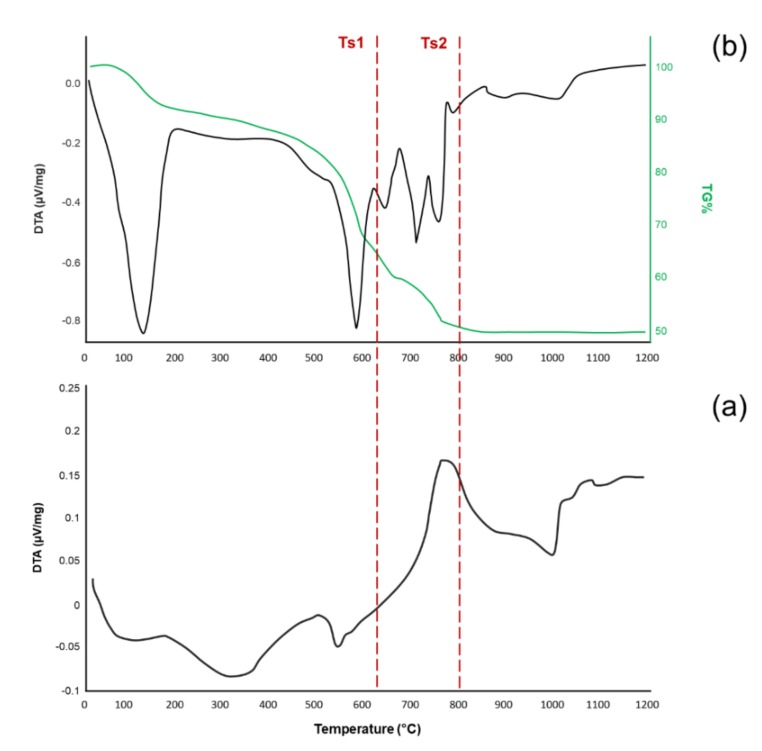
Thermal analysis results: DTA plot of MD-47.5B bioactive glass (**a**) and DTA-TGA plots of DG-120 sol-gel material before calcination, revealing a multi-peak trend and mass loss up to 800 °C (**b**).

The DTA thermograph of MD-47.5B (Figure 2a) revealed the characteristic features of glass. T_g_, T_x_ and T_c_ were identified at 550, 700 and 750 °C, respectively. Consistently to what observed in a previous report, the wide (T_x_ – T_g_) window of this glass (about 150 °C) allows the sintering to be performed without inducing any crystallization in the amorphous matrix [35]. This aspect is a clear advantage for scaffold manufacturing, as it allows mechanically resistant struts to be obtained while preserving the amorphous nature of the material and, thus, its bioactive potential in contact with body fluids.

As regards to the sol-gel-derived material, DTA and TGA were performed on the gel DG-120 in order to identify the most suitable calcination temperature for the final heating treatment (Figure 2b). The total mass loss up to 800 °C, assessed by TGA analysis, was about 50 wt %. It was possible to attribute most of the mass reduction to two different events, identified at about 110 and 580 °C, related to (i) the evaporation of residual water in the gel and (ii) the thermal decomposition of organic compounds and nitrates used as oxide precursors in the synthesis process, respectively. With respect to what was observed in the case of sol-gel 45S5 glass by Cacciotti et al. [20], who reported an endothermic peak associated to the decomposition of nitrates at 529 °C, our thermograph revealed a shift of the endothermic peak toward higher temperatures (from 529 to 580 °C). This can be attributable both to the higher complexity exhibited by 47.5B composition (compared to 45S5 system) and to the different heating rates used in the present study for thermal analyses. The exothermic peak at about 680 °C corresponds to the crystallization of combeite, as already confirmed by XRD analysis (this phase was detected in SG-800 but was not present in the SG-625). The endothermic peaks at about 712 and 755 °C can be attributed to further stages of the thermal decomposition of nitrates, especially sodium nitrate, as reported by Zheng et al. [37].

Unlike MD-47.5B, it was not possible to define a clear workability window (T_x_ – T_g_) and we decided to select two different calcination temperatures, T_s1_ (625 °C) and T_s2_ (800 °C), according to the following criteria:

- T_s1_ was chosen as the lowest calcination temperature able to ensure thermal decomposition of most nitrates and other organic compounds according to previous literature; this temperature was also below the crystallization temperature (exothermic peak centered at about 680 °C) and was very close to that used to sinter MD-47.5B products in previous works (600 °C) [35];
- T_s2_ corresponds to the maximum mass loss of the system and, thus, to the complete thermal stabilization of the material before reaching the melting temperature.

**Figure 3:**
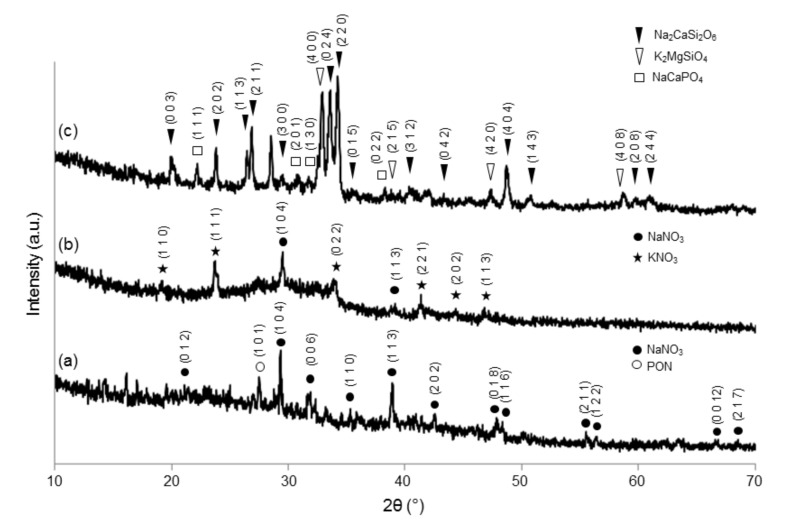
XRD pattern evolution upon thermal treatment of sol-gel 47.5B system at different stages of the synthesis process: DG-120 (**a**), SG-625 (**b**) and SG-800 (**c**).

The XRD pattern of MD-47.5B was previously reported by Fiume et al. [30]. It was characterized by an amorphous halo between 25° and 35°, which is typical of glassy silicate systems. Figure 3 shows the XRD patterns of the gel-derived materials at different stages of the synthesis process. The DG-120 XRD pattern (Figure 3a) revealed the presence of NaNO_3_, deriving from the precursor of Na_2_O introduced in the sol-gel process. This result was consistent with DTA curve interpretation, as the drying stage was performed at too low of a temperature (120 °C) to allow the thermal decomposition of nitrates in the gel.

Unlike MD-47.5B that was amorphous, SG-625 and SG-800 exhibited a certain crystallinity after calcination, as confirmed by XRD patterns reported in Figure 3b,c. The XRD pattern of SG-625 shows the typical appearance of a glass-ceramic material, where an amorphous halo centered between 25° and 35° is still clearly visible along with some diffraction peaks attributable to nitrates. Persistence of nitrates at 625 °C is consistent with both the DTA analysis (Figure 2b) and with the previous results reported by Zheng et al. [37], who detected the presence of NaNO_3_ in sol-gel 45S5 glass-ceramic after thermal stabilization at 700 °C. The XRD pattern of SG-800 exhibits sharp diffraction peaks indicating the clear development of multiple crystalline phases upon calcination.

The list of all the crystalline phases detected in SG-625 and SG-800, along with reference codes, formulas and crystal systems, are summarized in Table 1.

Interestingly, the major crystalline phase detected in SG-800 is the same (combeite-type Na_2_CaSi_2_O_6_) that was found by many researchers in the melt-derived 45S5 Bioglass^®^ after sinter-crystallization above 600 °C [38,39]. Sodium calcium silicate crystals (Na_2_Ca_2_Si_3_O_9_) were detected in MD-47.5B sintered at 750 °C [30], too, but this combeite-type phase was different compared to that observed in calcined SG-800.

**Figure 4:**
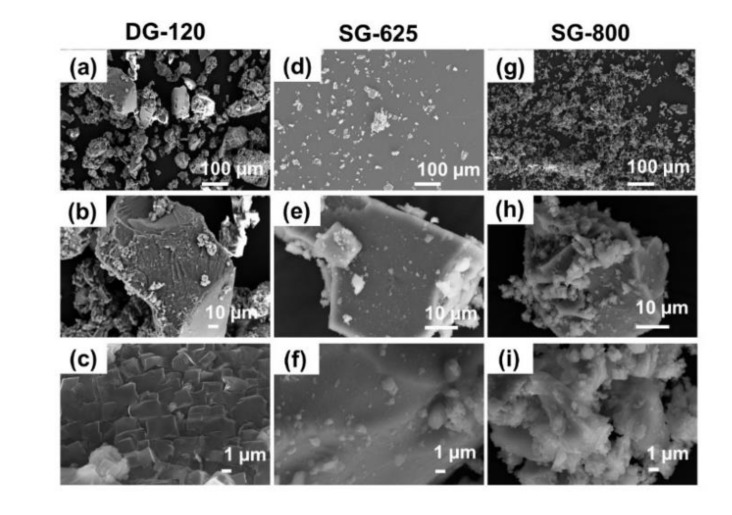
SEM morphological evaluation of DG-120 (**a**–**c**), SG-625 (**d**–**f**) and SG-800 (**g**–**i**) sol-gel-derived powders at different magnifications.

SEM morphological analyses of DG-120, SG-625 and SG-800 are shown in Figure 4 at different magnifications. Coarser granules (above 100 µm) were observed for the as-dried gel (DG-120), compared to the calcined sol-gel materials SG-625 and SG-800, as shown in Figure 4a–c. This was mainly due to the impossibility of effectively sieving the gel granules because of their high hygroscopic behavior and tendency to form aggregates. At higher magnification (Figure 4c), an ordered tile-like structure was observed. Finer particles with qualitatively more uniform size were observed for SG-625 and SG-800 as a direct result of the sieving process. In both cases, smaller particles tended to form aggregates on the surface of bigger ones, as clearly shown in Figure 4e,h.

**Figure 5:**
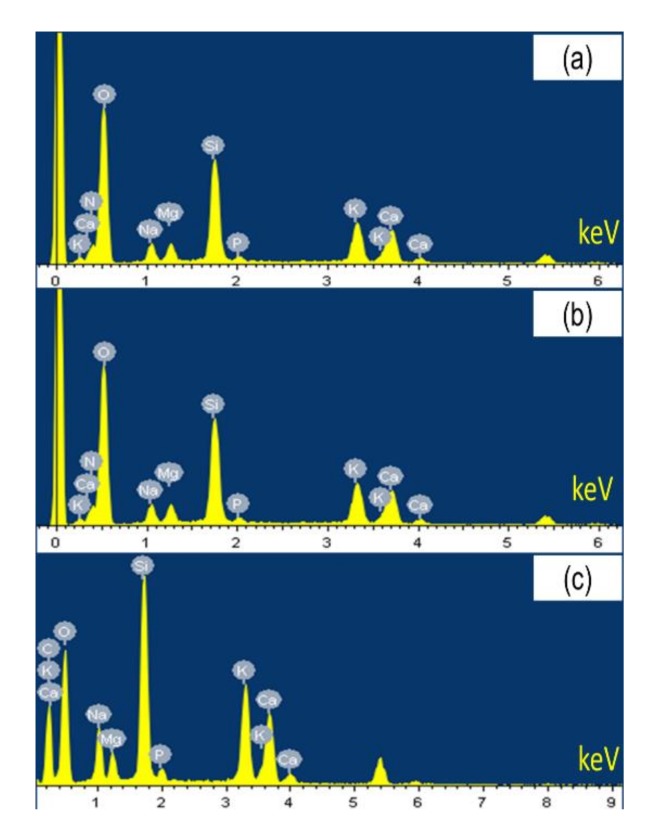
EDS compositional analysis performed on DG-120 (**a**), SG-625 (**b**) and SG-800 (**c**) sol-gel materials.

Consistently with the DTA and XRD results, EDS analysis performed on the as-dried gel and SG-625 (Figure 5a,b) confirmed the presence of nitrogen in the material composition deriving from the synthesis precursors, as a direct result of the low temperature used for the calcination process, while no nitrogen was detected in the glass-ceramic calcined at 800 °C (Figure 5c).

The BET results are summarized in Table 2. SG-625 and SG-800 exhibited higher SSA compared to MD-47.5B, which confirms the role played by the synthesis process (sol-gel vs. melt-quenching) on the textural properties of materials. The SSA tends to decrease by a factor 2 if the calcination temperature is increased from 625 to 800 °C because the materials nanoporosity—which is inherent of the sol-gel process—is reduced accordingly. A similar trend was observed for the pore volume, too. In general, the values of SSA of SG-625 and SG-800 are remarkably lower (from one to two orders of magnitude) than those observed in other silicate sol-gel biomaterials [14,40,41]: this might be attributed to the complex composition of the six-oxide system produced as well as to the development of crystalline phases, but further studies are required to better investigate this peculiar aspect in the future. Total pore volume values related to the three materials are consistent with the results discussed above.

**Figure 6:**
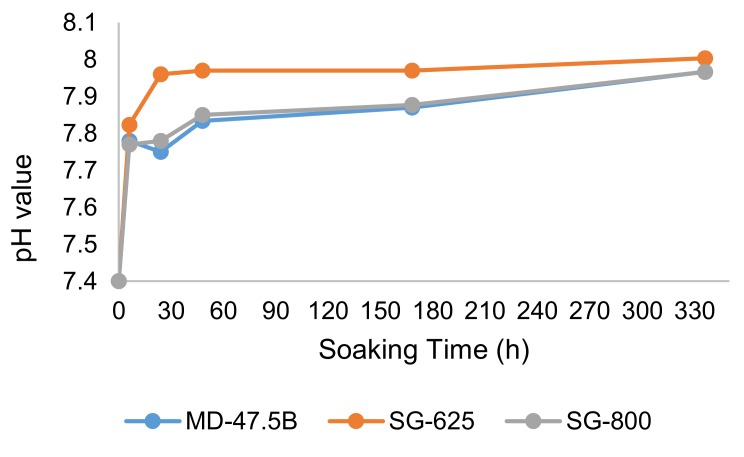
In vitro bioactivity tests: pH increase as a function of the soaking time in simulated body fluid (SBF).

The pH increase due to ion exchange between the materials and the solution upon in vitro bioactivity tests in SBF is plotted in Figure 6. Although no statistically significant differences were observed for the three systems, some observations could be made:

- The system showing the highest value of SSA was the one for which the highest pH increase was observed (i.e., SG-625), thereby suggesting a direct correlation between SSA and reactivity of the material in aqueous solution—in other words, the higher the SSA, higher the reactivity;
- For SG-625 glass-ceramic, the pH value stabilized after 48 h immersion with the achievement of a plateau at around 7.95, while a continuous increase up to two weeks was observed in the case of MD-47.5B and SG-800. This may suggest that most of the in vitro bioactivity reactions of SG-625 took place within two days from the beginning of the test; afterward, a chemical equilibrium was reached;
- SG-800- and MD-47.5B-related curves were comparable both in trends and in pH values, suggesting comparable reaction rates of the materials in SBF.

A moderate increase of pH (<8.0) toward alkalinity, like in the present case, is beneficial to osteoblasts [42]. On the contrary, other sol-gel bioactive materials reveal a tendency to markedly increase the pH of surrounding fluids due to high reactivity in SBF. In this regard, thermal treatment and, hence, crystallinity can play a role: for example, gel-derived 45S5 glass thermally treated at 700 °C led to a pH above 9.5 after seven days in SBF, while the same material calcined at 1100 °C (glass-ceramic) exhibited a lower reactivity (pH around 8.5 at one week) [20].

**Figure 7:**
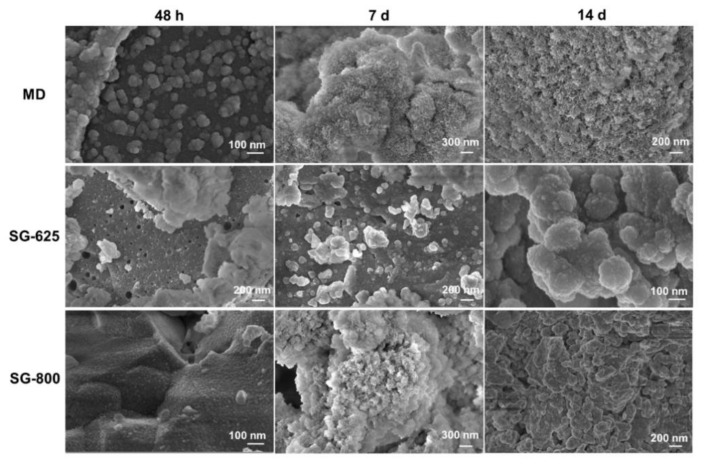
In vitro bioactivity tests: SEM analysis showing the surface evolution at different immersion time periods in SBF.

SEM morphological analyses after bioactivity tests in SBF (Figure 7) revealed an excellent apatite-forming ability of both melt-derived and sol-gel 47.5B-based materials, regardless of the production route used and calcination temperature. Calcium phosphate globular agglomerates were observed on the surface of MD-47.5B glass after just 48 h of soaking, confirming the high bioactivity of the positive control used in the present study.

After a two-day immersion in SBF, apatite-like structures were observed to form also on the surface of SG-625 and SG-800 glass-ceramics. A quite uniform, thin layer was observed on SG-800, while larger calcium phosphate agglomerates formed on SG-625. Despite the presence of crystalline phases in both sol-gel materials, the bioactivity mechanism was not inhibited. Nanopores in the range of 50–100 nm were observed on the surface of SG-625, suggesting a correlation between SSA values and this nanostructured feature.

After a two-week immersion in SBF, calcium phosphate coatings formed by nanostructured globular agglomerates, the morphology of which closely resembles that of hydroxyapatite, were observed on the surface of all the samples.

EDS measurements proved a progressive deposition of calcium and phosphorus on the surface of the materials. At the end of the test, atomic Ca/P ratios for MD-47.5B, SG-625 and SG-800 were 1.65, 1.63 and 1.48, respectively (average calculated on five sites per sample). The Ca/P ratios of MD-47.5B and SG-625 were quite close to that of stoichiometric hydroxyapatite (Ca/P = 1.67).

The formation of a hydroxyapatite layer in SBF is commonly recognized as the criterion to estimate the bioactive potential of (bio)materials. However, the relationship between in vitro results and in vivo behavior has been debated in the last decade. Kokubo and Takadama [33] reported convincing evidence that hydroxyapatite formation on the surface of a given material in SBF can actually be predictive of its bioactivity in vivo (i.e., bone-bonding ability). On the contrary, Bohner and Lamaitre [43] showed that this approach may be questionable due to some important limitations and there is still room for improvement. Currently, the scientific community recognizes the importance of in vitro tests in “inorganic” SBF to obtain a preliminary indication about bioactivity, although being aware that in vitro conditions can only roughly match those in the human body.

The formation of a hydroxyapatite layer on the surface of partially crystalline materials derived from a parent bioactive glass is not so obvious as devitrification can decrease bioactivity, as clearly demonstrated for melt-derived glass-ceramics in the SiO_2_–Na_2_O–CaO–P_2_O_5_ system [44]. The majority of bioactive silicate materials produced by the sol-gel route are in a glassy state; furthermore, it was shown that sol-gel glass-ceramics based on the 45S5 or 70S30C (70SiO_2_–30CaO mol %) systems retain a good apatite-forming ability regardless of the formation of crystalline phases [45]. The results achieved in the present work are consistent with these previous ones: the apatite-forming ability could be further improved in a future study by introducing a structure-directing agent in the sol-gel process (as already reported for simpler compositional systems [46]) to obtain mesoporous materials with ultrahigh SSA and, hence, higher reactivity.

## 4. Conclusions

Silicate materials with complex compositions based on a six-oxide system (47.5B) were successfully synthesized, for the first time, by the sol-gel route. Interestingly, while the melt-derived counterpart was fully amorphous, calcined sol-gel products are glass-ceramic. The complexity of the composition played a role in this regard as conventional binary or ternary sol-gel glasses are known to be typically amorphous. The method of production has a clear effect on the textural properties, as the SSA of sol-gel 47.5B-based materials was from two to four times higher than that of melt-derived glass. However, the SSA values of sol-gel 47.5B-based materials were drastically lower than those detected in sol-gel glasses with simpler formulation (few m^2^/g vs. tens/hundreds of m^2^/g); specifically, an increase of calcination temperature was associated to a decrease of SSA. Interestingly, sol-gel 47.5B-based glass-ceramics still exhibited a promising bioactive potential regardless of the formation of crystalline phases, the presence of which, instead, is often associated with a dramatic decrease of apatite-forming ability in melt-derived materials. In the future, in vitro studies with appropriate cell lines will be carried out in order to assess the cytocompatibility of the materials here investigated.

## Figures and Tables

**Table 1:** Crystalline phases detected in SG-625 and SG-800 sol-gel materials.

Detected in:	Phase Name	Reference Code	Formula	Crystal System
SG-625	Niter	01-071-1558	KNO_3_	Orthorhombic
	Nitratine	00-036-1474	NaNO_3_	Rhombohedral
SG-800	Sodium calcium silicate (combeite-type)	01-077-2189	Na_2_CaSi_2_O_6_	Rhombohedral
	Potassium magnesium silicate	00-048-0900	K_2_MgSiO_4_	Orthorhombic
	Rhenanite	00-029-1193	NaCaPO_4_	Orthorhombic

**Table 2:** Brunauer–Emmett–Teller (BET) analysis results.

Material	Class	Calcination Temperature (°C)	SSA (m^2^/g)	Pore Volume (cm3/g)
MD-47.5B	Glass	As-quenched	0.6379	0.001304
SG-625	Glass-ceramic	625	2.2330	0.016708
SG-800	Glass-ceramic	800	1.2307	0.002727
